# Editorial: Immunosenescence in the cancer microenvironment

**DOI:** 10.3389/fimmu.2023.1161110

**Published:** 2023-02-27

**Authors:** Annibale Alessandro Puca, Elena Ciaglia

**Affiliations:** ^1^ Department of Medicine, Surgery and Dentistry “Scuola Medica Salernitana”, University of Salerno, Salerno, Italy; ^2^ Cardiovascular Research Unit, IRCCS MultiMedica, Milan, Italy

**Keywords:** senescence, therapy resistance, immune-suppression, aberrant crosstalk, biomarker

Senescence is thought to be a major barrier to the tumor formation, as it limits the replicative potential of many tumors including melanoma, hepatocellular carcinoma, and medulloblastoma. In glioblastoma, despite the promising therapeutic effect of temozolomide or etoposide, the therapy-induced cancer senescent cells remain metabolically active, and this brings attention to the potential side effects of cancer cell senescence, mainly evasion from therapy-induced senescence and resistance occurrence. Further the spreading of senescence to the neighboring cells, mainly immune cells can both enhance and dampen antitumor response.

Based on these premises, the understanding of senescence signature in cancer microenvironment may be a valuable indicator for patients’ prognosis and therapy response.

The present collection includes original contributions highlighting the role of the cellular senescence and the peculiar immune status in osteosarcoma, peritoneal carcinomatosis, gastric and color cancer, cell renal cell and hepatocellular carcinomas, gliomas and in a set of bladder and endometrial cancers with particular attention to the senescence-based molecular features and the perturbation of the immune profile of the tumor microenvironment (TME).

The selected manuscripts took advantage of the rapid progress in the development of next-generation sequencing (NGS) technologies and in available bioinformatics tools which in recent years are greatly facilitating both the basic science and the clinical approaches by providing many valuable insights into complex cancer genomics.

The Cancer Genome Atlas (TCGA) and Gene Expression Omnibus (GEO) database were the main source of multi-omics data and clinical informations used by the authors to describe several score models, which are not only a robust prognostic indicator for patients’ prognosis but also provides a new reference basis for personalized treatment selection.

- Based on the widespread application of single-cell RNA sequencing (scRNA-seq) technology Zhang et al. reported that aging-related MS4A6A is overexpressed in glioma tissue at both transcriptional and protein levels, and it is related to the degree of suppressing macrophage infiltration and to the significant decrease in overall survival (OS).
- Through a combination of bulk-sequencing and single-cell sequencing technology, Tu et al. demonstrated that a peculiar subpopulation of tissue-resident macrophages (*C1Q*+ TAMs), which often expresses *CD206*, *HLA-DR*, *SEPP1*, *FOLR2*, and *APO* associated with the overall survival of osteosarcoma patients and that most of the high expression of immune infiltration-related genes links to better survival. As authors stated, this study provided evidence of how immune cells influence prognosis in osteosarcoma and that *C1Q*+ TAMs can be therapeutic target cells to improve the osteosarcoma treatment.

With the advancement of next-generation sequencing technologies, RNA modifications have gained much attention because they participate in several physiological and pathological processes.

- A comprehensive analysis of RNA sequencing (RNA-seq) data and clinical information using TCGA and GEO databases led Zhao et al. to discover that the RNA methylation, e.g. N^1^-methyladenosine (m1A) modifications, have a potential role in the prognosis of hepatocellular carcinoma (HCC) as these modifications clearly associated with different overall survival, TNM stage and tumor immune microenvironment (TIME) characteristics, mainly impacting prognosis and therapy response.
- Similarly Zhang et al. assessed the RNA 5-methylcytosine (m5C) modification pattern for individual gastric cancer patients and the association with the degree of immune infiltration and the most favorable prognosis in terms of tumor recurrence and immune checkpoint sensitivity.
- Lu et al. applied a novel modelling algorithm to construct a pyroptosis-related lncRNA signature in the bladder cancer. Long noncoding RNAs (lncRNAs) contribute to regulating immune microenvironment in many diseases and here the authors disclosed that this pyroptosis-related lncRNA risk model possessed good prognostic value, and the ability to predict the outcome of immunotherapy. Mechanism analysis revealed that high risk group was characterized by a higher proportion of M0 macrophages and M2 macrophages than those in the low-risk score group. However, the proportion of CD8+ T cells was significantly lower in the high-risk score group. Patients with lower risk score were characterized by higher immune checkpoint gene expression and Tumor Mutation Burden (TMB) and display a better response to immunotherapy and chemotherapy compared with patients with high-risk score.
- Similarly Chen et al. analyzed the role of Programmed Cell Death-related lncRNAs in the prognosis of colon cancer patients and their correlations with clinicopathological characteristics, the TME, the immune checkpoint genes, TMB and both immunotherapy and chemotherapeutic drug sensitivity, mainly focusing on the biological functions of the lncRNA U62317.4
- Starting from EMT-related genes (ERGs) expression and clinical data from TCGA Liu et al. develop Epithelial-mesenchymal transition (EMT)-related prognostic signature for endometrial cancer patients among which the low-risk group showed more immune activities, higher TMB and better therapy response. On the contrary, the high-risk group had higher m6A RNA expression, microsatellite instability level and stemness indices.
- Interestingly, Zhou et al. by screening RNA-seq data of the TCG-Kidney renal clear cell carcinoma (KIRC) cohort identified senescence subtypes in tumor samples. Using the adaptive lasso regression, they established a senescence score and demonstrated it was negatively correlated with immune infiltration. Furthermore, signatures from different gene expression profiles suggested that the senescence program is closely correlated with CAFs of the tumor stroma, self-limited antitumor immunity, and drug sensitivity. Consequently, a senescence score was applied to predict patients’ prognosis and to properly distinguish anti–PD-1 responders in Clear Cell Renal Cell Carcinoma -ccRCC.
- At the same way, Sun et al. identified a four-cell-senescence-regulator-gene prognostic index in bladder cancer and investigated its relationship with TMB, the immune landscape of TME and response to immunotherapy and chemotherapy.
- A computational approach by Zhu et al. offered for the first time interaction between aging-associated genes in ccRCC and remodeling of the TME, providing a novel insight into the molecular drivers underlying ccRCC initiation and development.

Of note, the special issue also reported the discover of valuable biomarkers such as the predictive value of CD93 in pan-cancer. CD93 is a transmembrane receptor that is mainly expressed on endothelial cells and macrophages and Guo et al. here characterized the association between its mRNA level and immune scores, stromal scores, immune infiltrates, immune checkpoints, and prognosis in most cancer which might suggest to targeting it as a promising therapeutic strategy.

Interestingly in a murine orthotopic transplantation model for peritoneal carcinomatosis (PC), Braumüller et al. showed that the tumor cells senescence in PC is associated with a senescence-associated secretory phenotype (SASP) influencing the tumor microenvironment of PC. SASP factors can induce a senescence phenotype in neighboring cells mainly determing immunosenescence in the TME of PC. According to the focus of our special issue, these results provide a new immunoescape mechanism in PC of colorectal cancer explaining the resistance of PC to known chemo- and immunotherapeutics. Therefore, senolytic approaches may represent a valuable therapeutic opportunity to threat this terminal stage of CRC.

Finally Chen et al. reviewed the latest research progress in the immune microenvironment and strategies related to immunotherapy for colorectal cancer for the proper selection of treatment strategies for CRC patients.

Overall, we hope that all manuscripts enclosed in this Research Topic may be of interest for the readership who constantly look for useful references to optimize cancer prevention, early detection, prognosis as well as therapy.

## Author contributions

All authors listed have made a substantial, direct, and intellectual contribution to the work and approved it for publication.

